# Short-Term Consumption of Hot Beverages in Polystyrene Cups and Early Biomarkers of Biological Effect: A Single-Arm Longitudinal Human Biomonitoring Pilot Study

**DOI:** 10.3390/jox16030084

**Published:** 2026-05-15

**Authors:** Iman Al-Saleh, Ghofran Al-Qudaihi, Yara Aljerayed, Kafa Abuhdeeb, Rola Elkhatib, Hissah Alnuwaysir, Mashael Alsubaie, Norah Alotaibi

**Affiliations:** Environmental Health Program, Research Centre (MBC # 03), King Faisal Specialist Hospital & Research Centre, Takhassosi Street, P.O. Box 3354, Riyadh 11211, Saudi Arabia

**Keywords:** styrene exposure, polystyrene food-contact materials, human biomonitoring, comet assay, early biomarkers of biological effect, consumer exposure

## Abstract

Styrene, a constituent of polystyrene food-contact materials, can migrate into hot beverages, but data on short-term consumer exposure and associated biological responses remain limited. In this single-arm longitudinal human biomonitoring pilot study, 40 healthy adults consumed tea or coffee daily in Styrofoam cups for approximately two weeks. Biomarkers were measured at baseline, day 6, and day 11, including urinary mandelic acid (MA) and phenylglyoxylic acid (PGA), salivary malondialdehyde (MDA), comet assay parameters in peripheral blood lymphocytes, and micronucleus (MN) frequency in buccal cells. Measured styrene migration into beverages ranged from 3.3 to 7.1 μg/L, below the World Health Organization guideline value. Urinary metabolites and salivary MDA showed substantial interindividual variability and no consistent temporal pattern. In contrast, generalized estimating equation models showed progressive increases in comet assay indicators over the exposure period. Tail intensity and tail moment increased over time, with stronger changes among participants consuming two cups daily. MN frequency did not change significantly. These findings suggest that repeated short-term consumption of hot beverages in polystyrene cups was associated with modest changes in selected early biomarkers of biological effect under consumer-use conditions. The results should be interpreted cautiously in light of the modest sample size, short follow-up, and absence of more specific mechanistic endpoints, but they support further study of repeated low-level exposure to food-contact materials.

## 1. Introduction

Styrene is a widely used industrial chemical, primarily employed in the manufacture of polystyrene plastics and resins. Occupational exposure to styrene is well documented and has been linked to a variety of toxic effects, including ototoxicity, neurotoxicity, hepatotoxicity, reproductive and developmental effects, and increased risk of cancer [1,2,3,4,5,6,7]. The International Agency for Research on Cancer (IARC) classified styrene and its primary reactive metabolite, styrene-7,8-oxide, as probably carcinogenic to humans (Group 2A) [8]. After absorption, styrene undergoes extensive hepatic metabolism via cytochrome P450–mediated oxidation to styrene-7,8-oxide, which is subsequently hydrolyzed and further metabolized via several pathways [9]. Styrene-7,8-oxide is considered the primary reactive intermediate of styrene metabolism and can undergo further hydrolysis and oxidation to form styrene glycol, mandelic acid (MA), and phenylglyoxylic acid (PGA), while minor pathways may involve conjugation with glutathione or glucuronic acid [8,10,11]. Therefore, urinary MA and PGA are commonly used as integrated biomarkers of styrene exposure [12], whereas styrene-7,8-oxide is more directly relevant to genotoxic potential [13]. The two primary urinary metabolites, MA and PGA, are formed by the oxidative degradation of styrene-7,8-oxide and account for approximately 60–70% of the styrene dose excreted in urine [14,15,16]. Due to their abundance and relative stability, MA and PGA, either measured individually or as their sum Σ(MA + PGA), are widely used as biomarkers of styrene exposure in both occupational and environmental studies [12,17].

Styrene has been reported to exert genotoxic potential primarily through metabolic activation to styrene-7,8-oxide, which can form DNA adducts and induce mutations in vitro [18]. In addition to DNA adduct formation, styrene-7,8-oxide has been associated with DNA strand breaks and chromosomal damage, supporting its role as a key mediator of styrene-related genotoxicity [19]. Overall, the evidence suggests that this reactive metabolite is primarily responsible for styrene’s genotoxicity, with consistent positive findings in vitro but limited confirmation in vivo [20]. Human studies among styrene-exposed workers have shown increased DNA adducts, chromosomal aberrations, and elevated comet assay parameters, although results vary depending on exposure intensity, co-exposures (e.g., acetone), genetic polymorphisms, and biomarker sensitivity [21,22].

Although occupational health effects of styrene are well studied, exposure in the general population may also occur through multiple pathways, including ambient air, cigarette smoke, occupational settings involving styrene or aromatic vinyl monomers, and combustion-related emissions, in addition to migration from food and drink containers [23]. Migration of styrene from polystyrene packaging into beverages and foods has been repeatedly demonstrated, especially when in contact with heat, fat, alcohol, or acidic content [24,25,26,27,28]. In many countries, disposable Styrofoam cups are widely used for hot beverages, raising concerns about chronic low-dose exposure to these materials. Regulatory agencies, including the US Environmental Protection Agency and the Food and Drug Administration, have set limits on styrene in water and food; however, restrictions remain limited due to insufficient evidence on health risks at low environmental exposures.

Recent advances in biomonitoring have highlighted saliva and exfoliated buccal cells as promising noninvasive matrices for assessing chemical exposures and genotoxicity [29,30,31]. Saliva has shown strong correlations with blood styrene levels in occupational settings [32], but studies in non-occupational populations are scarce. Genotoxic effects of styrene have been demonstrated in occupational cohorts using biomarkers such as the comet assay, micronucleus (MN) assay, sister chromatid exchange, and chromosomal aberrations [33,34]. Buccal MN assays are particularly attractive as they directly assess epithelial cells at the first point of contact with ingested styrene.

To date, little is known about whether short-term consumption of hot beverages in Styrofoam cups leads to measurable internal exposure and detectable early biological responses in healthy adults. Therefore, this study evaluated styrene-related exposure biomarkers and selected response markers in participants who consumed hot beverages from Styrofoam cups for two weeks. Specifically, we measured urinary styrene metabolites, salivary MDA, comet assay parameters in peripheral blood lymphocytes, and MN frequency in buccal cells. Although DNA adduct measurements were originally planned, they could not be completed because of instrument failure. Accordingly, this study should be viewed as a single-arm longitudinal human biomonitoring pilot investigation of early biomarkers of biological effect under consumer-use conditions.

## 2. Materials and Methods

### 2.1. Study Design and Participants

This single-arm prospective longitudinal study included 40 healthy adult volunteers, primarily hospital employees rather than hospitalized patients, recruited in a workplace setting. During recruitment, baseline data were initially obtained from 41 healthy adult volunteers who provided written informed consent between 2019 and 2021; one individual did not contribute complete study data and was therefore excluded from subsequent analyses. Outcome-specific sample sizes varied across biomarkers due to missing measurements. During the intervention period, participants consumed hot beverages using the same locally supplied Styrofoam cup type routinely offered by the hospital cafeteria, which was also evaluated in the styrene migration experiment. Participants were instructed to maintain their usual diet and daily activities during the intervention period, with the only protocol-mandated exposure being the consumption of one or two hot beverages daily in the provided Styrofoam cups. For this study, hot beverages were defined as tea or coffee consumed in the provided Styrofoam cups; water, cold beverages, juices, and other drinks packaged in plastic bottles were not part of the protocol-mandated exposure. Inclusion criteria were as follows: (i) healthy adults aged 22–42 years; (ii) both sexes; (iii) absence of current chronic illness or ongoing medication use at the time of enrollment; (iv) non-smoking status; (v) not wearing dentures; and (vi) refraining from the use of Styrofoam cups or containers for at least seven days before study entry. Participants with known occupational exposure to styrene or related solvents were not eligible. During the intervention, participants were instructed to consume one or two hot beverages daily in the provided Styrofoam cups. Adherence to the washout and intervention procedures was based on participant instruction and self-report rather than direct observation or daily exposure diaries. Body mass index (BMI) was not an exclusion criterion unless accompanied by clinical instability. One participant had a BMI of 13.7 kg/m^2^; she was an adult, clinically stable at enrollment, and experienced no adverse events related to blood collection.

At the outset, demographic and lifestyle data were collected from each participant, including age, sex, occupation, and previous use of Styrofoam cups or food containers. Baseline samples of urine, saliva, blood, and buccal cells were then obtained. This prospective approach allowed us to monitor changes in exposure biomarkers and selected early biological response markers over time. Follow-up urine, saliva, blood, and buccal cell samples were collected on days 6 and 11 to assess potential effects of repeated daily consumption of hot beverages from Styrofoam cups.

**Figure 1:**
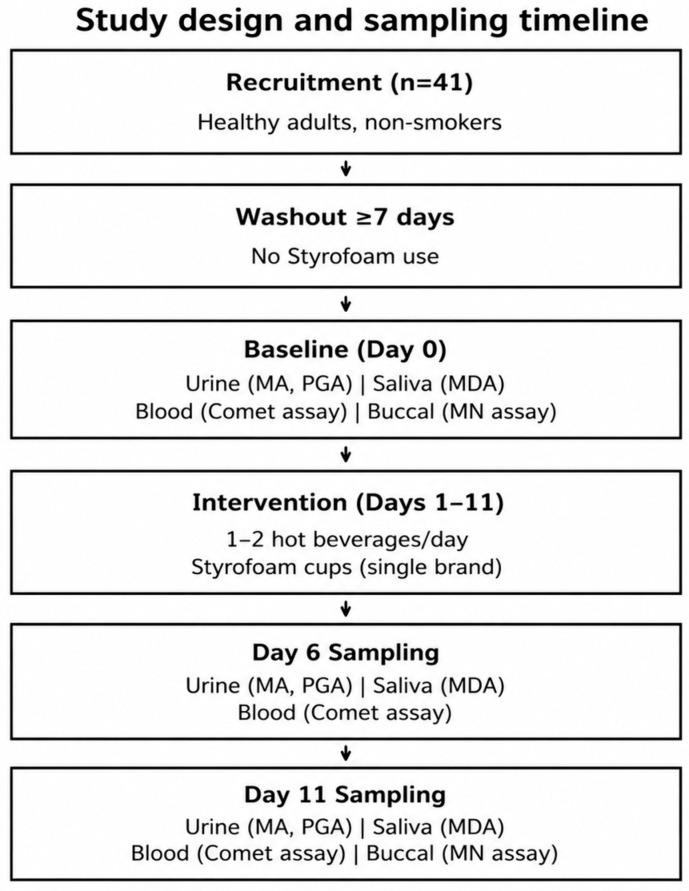
Schematic overview of the study design, exposure period, and biological sampling timeline. Healthy adult participants underwent a washout period prior to baseline sampling, followed by daily consumption of hot beverages in Styrofoam cups over an 11-day intervention period. Biological samples were collected at baseline, day 6, and day 11, and analyzed for styrene exposure biomarkers (MA and PGA) and biomarkers of biological effect, including oxidative stress (MDA), comet assay parameters, and MN frequency. Styrene migration testing was conducted separately using the same Styrofoam cup type.

A schematic overview of the study design, exposure period, and biological sampling timeline is presented in Figure 1.

### 2.2. Ethical Approval

All procedures involving human participants were conducted in accordance with institutional guidelines and the World Medical Association Declaration of Helsinki. The study protocol was reviewed and approved by the Research Ethics Committee of King Faisal Specialist Hospital and Research Centre (RAC #2150028), with formal ethical approval granted on 29 May 2019.

Written informed consent was obtained from all participants. Privacy and confidentiality were strictly protected, and all personal identifiers were removed from data and biological samples in accordance with institutional policies.

The study involved the collection of urine, saliva, buccal cells, and venous blood from healthy adult volunteers. No human organs or tissues were used, and no materials were obtained from executed prisoners or prisoners of conscience.

### 2.3. Styrene Migration Testing Method

Styrene migration from expanded polystyrene (Styrofoam) cups was evaluated using a headspace solid-phase microextraction (HS-SPME) method coupled with gas chromatography–mass spectrometry (GC–MS). Ten Styrofoam cups per beverage (10 filled with tea and 10 filled with coffee), all from the same locally supplied cup type routinely used by the hospital cafeteria, were analyzed after 60 min of contact under ambient laboratory conditions to simulate hot beverage consumption. The 60 min contact time was selected as a conservative exposure scenario, consistent with previous studies demonstrating increased styrene migration with longer contact durations and standardized migration-testing conditions [35]. After the contact period, 10 mL of each beverage was transferred into 20 mL glass SPME vials for analysis. Cup type and size and beverage volume were consistent across experiments, and all samples were handled identically under ambient laboratory conditions. Beverages were freshly prepared, poured immediately into the cups, and left uncovered and unstirred during the contact period. Beverage temperature was not instrumentally controlled but followed the same preparation and cooling procedures for both tea and coffee. This standardized approach represents a conservative exposure scenario and may overestimate typical real-world consumption conditions, as individual drinking duration, exact temperature, and contact time were not directly measured.

Styrene standards were prepared from analytical-grade styrene (Sigma-Aldrich, St. Louis, MO, USA) in HPLC-grade methanol (Fisher Scientific, Waltham, MA, USA). A primary stock solution (1 μg/mL) was prepared, and calibration standards were generated by spiking deionized water to obtain final concentrations ranging from 1 to 16 ng/mL. Recovery samples (1.5, 3, and 6 ng/mL) were prepared and analyzed alongside study samples. Method performance was evaluated using spiked recovery samples (1.5, 3, and 6 ng/mL), yielding recoveries ranging from 96% to 104%, with relative standard deviations (RSDs) between 2% and 6%, indicating good accuracy and precision of the analytical method. The method detection limit was 0.5 ng/mL.

Analyses were performed using an Agilent 6890N gas chromatograph coupled to an Agilent 5973 inert mass selective detector (Agilent Technologies, Santa Clara, CA, USA), equipped with a DB-5MS capillary column (30 m × 0.25 mm i.d., 0.25 μm film thickness; Agilent J&W Scientific, Folsom, CA, USA). Headspace extraction was carried out using a 65 μm PDMS/DVB StableFlex SPME fiber (Supelco, Bellefonte, PA, USA) mounted on a CTC Analytics PAL autosampler. Helium (99.999% purity) was used as the carrier gas. Quantification was based on external calibration using the prepared aqueous standards.

Measured styrene concentrations in beverages were expressed as μg/L for reporting and subsequent contextual comparison with existing guideline values. Styrene concentrations were used to derive an estimated daily intake based on beverage consumption.

### 2.4. Sample Collection and Storage

- Urine: ~20 mL collected in sterile containers at baseline, day 6, and day 11; stored at −20 °C.
- Saliva: 5 mL of unstimulated saliva collected after mouth rinsing; centrifuged at 4 °C for 10 min, supernatant stored at −80 °C.
- Buccal cells: Collected using a cytobrush rotated against the inner cheek and suspended in buffer (0.1 M EDTA, 0.01 M Tris-HCl, 0.02 M NaCl, pH 7.0).
- Blood: 5 mL venous blood collected in EDTA tubes; aliquots stored at −80 °C for comet assay analysis.

### 2.5. Analytical Methods

MA and PGA in urine were measured using a Chromsystems reagent kit (Chromsystems Instruments and Chemicals GmbH, Munich, Germany), in strict accordance with the provided instructions. For analysis, a 20 μL aliquot of the urine sample was injected onto the Waters Alliance HPLC 2695 system, which was connected to an ultraviolet detector set at 207 nm (Waters Corp., Milford, MA, USA). The chromatographic mobile phase was supplied as part of the Chromsystems reagent kit (Chromsystems Instruments and Chemicals GmbH, Munich, Germany).

The quantification of MDA in saliva samples was performed using the Waters Alliance High-Performance Liquid Chromatography (HPLC) 2695 system, paired with a multi-fluorescence detector (waters, Milford, MA, USA), Model 2475. The chromatographic mobile phase consisted of acetonitrile (Fisher Scientific, Waltham, MA, USA) and 50 mM potassium phosphate buffer (pH 7.0; 30:70, *v*/*v*), used in isocratic mode. This method, as described by Al-Saleh et al. [36], was validated using two commercial freeze-dried plasma reference materials (ClinChek^®^ Plasma Control lyophilized Level I and II, lot No. 1099) sourced from Recipe Chemical and Instruments, Munich, Germany. The results were consistent with the manufacturers’ specified concentrations, attesting to the method’s accuracy. The lower detection limit for saliva MDA was determined to be 0.069 nmol/mL.

Peripheral blood lymphocytes were isolated for the comet assay, conducted according to the standardized protocol by Singh et al. [37], with slight modifications [38]. Briefly, following lysis, slides were placed in alkaline electrophoresis buffer (300 mM NaOH and 1 mM EDTA; pH > 10) for 20 min to allow DNA unwinding prior to electrophoresis. Electrophoresis was performed at 25 V (approximately 0.8 V/cm) and 300 mA for 30 min under chilled conditions. Slides were subsequently neutralized, washed, fixed in 70% ethanol, air-dried, and stained with SYBR Green solution (Trevigen, Gaithersburg, MD, USA) according to the manufacturer’s recommended concentration. Analysis was performed on 50 cells per participant, distributed across two slides (25 cells per slide), using the Comet Assay IV software and a monochrome CCD IEEE 1394 FireWire video camera (Perceptive Instruments, Halstead, UK). Comet scoring was performed by trained laboratory personnel using the same scoring procedure across all time points. Values were reviewed for implausible or technically invalid measurements, and observations were excluded only when sample quality or missing data prevented reliable analysis. Blinding was not implemented, which is acknowledged as a limitation. This limitation is further addressed in the Discussion. Although some methodological recommendations suggest scoring ≥ 100 comets per individual to maximize statistical sensitivity, foundational studies [37] and subsequent expert consensus emphasize methodological consistency and appropriate statistical analysis rather than a fixed universal cell count [39]. In the present longitudinal repeated-measures design, each participant served as their own control, and analyses were performed using generalized estimating equations to account for within-subject correlation; therefore, scoring 50 comets per time point was considered sufficient to detect relative temporal changes in DNA damage. A fluorescence optical microscope (Eclipse Ti-E; Nikon, Tokyo, Japan), equipped with excitation (465 nm) and barrier (595 nm) filters, was used for image analysis at 20× magnification. Parameters such as head length (HL), tail length (TL), head intensity (HI), tail intensity (%DNA in tail), and tail moment (TM) were quantified. TM was prespecified as the primary endpoint for statistical analysis of DNA strand breaks, because it provides more stable estimates of DNA damage, exhibiting lower variability and greater uniformity in quantile dispersion compared with other comet assay parameters in human biomonitoring studies [40]. Tail intensity (%DNA in tail), recognized in MIRCA recommendations as a primary comet assay descriptor, was also evaluated and reported as an important comet assay parameter, while TM was retained as the prespecified primary endpoint [41].

The MN assay was conducted on buccal cells using the modified protocol of Thomas et al. [42]. Exfoliated buccal cells were collected by gently rotating a sterile cytobrush against the inner cheek mucosa and immediately placed in buccal cell buffer. Samples were washed by centrifugation, filtered to remove aggregates, and cytocentrifuged onto microscope slides. Slides were air-dried and fixed in ethanol:acetic acid (3:1) prior to Feulgen–Schiff staining and counterstaining, following the HUMNxL protocol [42]. A minimum of 1000 differentiated buccal epithelial cells per participant were scored for each sampling point under a Nikon Ti2 transmitted light and fluorescence microscope, according to the criteria established for exfoliated buccal epithelial cells within the HUMNxL project [42,43]. While the HUMNxL protocol recommends scoring up to 2000 differentiated buccal cells to maximize sensitivity [42], several published human biomonitoring studies have evaluated 1000 cells per subject and expressed MN frequency per 1000 cells [44,45]. In the present single-arm longitudinal design, 1000 differentiated cells per participant were scored, and MN frequency was expressed as the number of micronucleated cells per 1000 cells analyzed. One participant was excluded from this analysis because the buccal sample lacked sufficient cells. Therefore, the MN assay included 39 participants. The assay was performed only at baseline and day 11; however, because buccal epithelial cell turnover may extend up to 21–28 days, day 11 sampling may have been too early to fully capture exposure-related MN formation [42,43,46].

Urinary cotinine was measured by ELISA (Bio-Quant, San Diego, CA, USA) to verify non-smoking status (sensitivity: 1 μg/L), and urinary creatinine was measured using a commercial assay (Oxford Biomedical Research, Oxford, MI, USA) for metabolite normalization.

Biological samples (urine, saliva, blood, and buccal cells) were collected at baseline, day 6, and day 11. Urinary MA and PGA, salivary MDA, comet assay parameters in lymphocytes, and MN frequency in buccal cells were determined using standard protocols. Secondary outcomes included %DNA in tail, TL, urinary MA and PGA, salivary MDA, and MN frequency.

### 2.6. Statistical Analysis

Data were analyzed using SPSS Statistics version 26.0 (IBM Corp., Armonk, NY, USA). Continuous variables were summarized as mean ± SD or median (range), and categorical variables as counts and percentages. Bivariate associations were examined using Spearman’s rank correlation for continuous predictors and Mann–Whitney U tests for categorical predictors (Table S2). Variables showing significant or borderline associations (*p* < 0.10) were considered for inclusion in multivariable modeling.

Longitudinal changes in biomarkers were assessed using generalized estimating equations (GEE), which account for correlated repeated measures and missing data. An exchangeable correlation structure was specified. Pairwise comparisons were conducted between baseline, day 6, and day 11, as well as between exposure groups (one vs. two cups/day). To control for multiple testing, a Bonferroni adjustment was applied, and only adjusted *p*-values are reported. All models were adjusted for age, sex, and body weight.

Results are presented as regression coefficients (β) with corresponding 95% confidence intervals (CI) and Bonferroni-adjusted *p*-values. Because of model coding, negative β coefficients for baseline comparisons indicate higher biomarker values at follow-up than at baseline. For comparisons by daily cup consumption, positive β coefficients indicate higher biomarker values among participants consuming two cups per day compared with one cup per day. This coding direction explains why some coefficients may appear negative even though raw data, such as boxplots, show increasing trends.

In addition, descriptive percentage changes were calculated as follows:

Percentage change (%) = [(Value follow-up − Value baseline)/Value baseline] × 100

Percentage changes were calculated descriptively from estimated marginal means (EMMs), using baseline values as the reference for temporal comparisons and the one-cup group as the reference for two-cup vs. one-cup comparisons. This allowed us to present GEE results as relative changes, complementing the β coefficients and CIs. A *p*-value < 0.05 after Bonferroni adjustment was considered statistically significant. Post hoc power calculations were not performed, as observed power is mathematically determined by the *p*-value and does not provide additional interpretive value beyond effect estimates and confidence intervals [47].

## 3. Results

### 3.1. Participant Characteristics

Baseline characteristics were available for 41 participants (12 males, 29 females). The median age was 30.8 years (range 22–42) and the median BMI was 25.3 kg/m^2^ (range 13.7–37.7). Twenty-one participants were Saudi nationals, and 20 were from other nationalities. The non-Saudi participants included Filipino, Indian, Sudanese, Yemeni, and Eritrean participants; however, subgroup analyses by nationality were not performed due to small numbers in each group. Three participants reported a past medical history (atopic dermatitis, appendicitis, and open-heart surgery); however, all were clinically stable, symptom-free, and not receiving chronic medication at the time of enrollment. Six participants reported using Styrofoam for food storage on an occasional or rare basis. Regarding beverage consumption during the study, 19 participants reported drinking once a day, while 22 participants reported drinking twice a day. Coffee was the most commonly consumed beverage during the intervention (*n* = 24), followed by tea (*n* = 10) and both (*n* = 7). Although all participants self-reported as nonsmokers, urinary cotinine levels > 1 μg/L were detected in 18 individuals, suggesting possible secondhand smoke exposure. The general characteristics are presented in Table 1.

### 3.2. Styrene Concentrations and Estimated Intake 

Styrene concentrations in beverages prepared in Styrofoam cups ranged from 3.3 to 7.1 μg/L, remaining below the WHO guideline limit of 20 μg/L [10]. Based on these measured concentrations and a standardized cup volume of 8 oz (240 mL), the estimated styrene intake per cup ranged from 0.93 to 1.45 μg, corresponding to an estimated daily intake of 0.93–1.45 μg/day for participants consuming one cup per day and 1.86–2.89 μg/day for those consuming two cups per day.

### 3.3. Urinary Metabolites and Oxidative Stress

**Figure 2:**
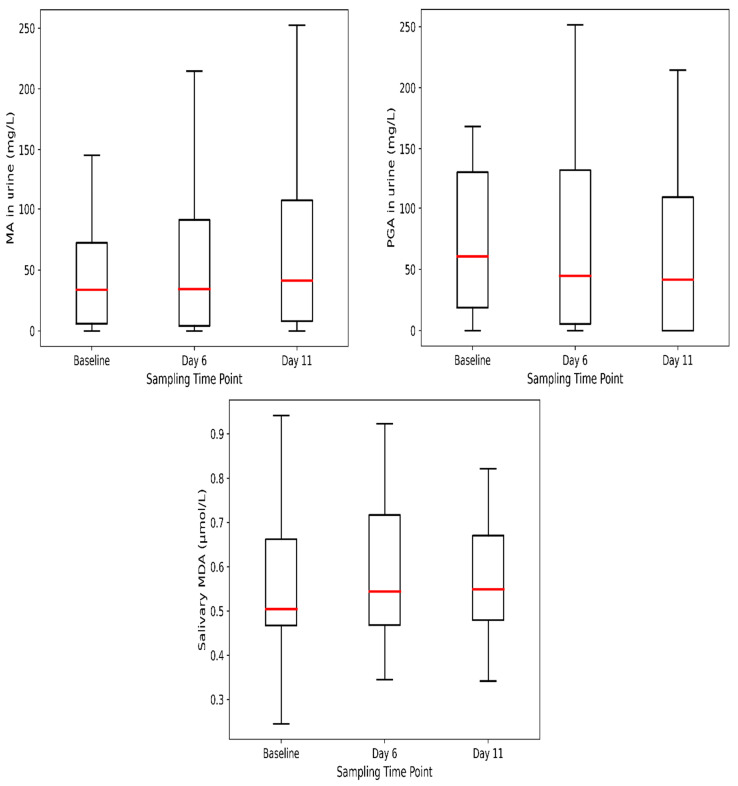
Boxplots showing concentrations of urinary MA, PGA, and salivary MDA at baseline, day 6, and day 11. Boxes represent the interquartile range (IQR), the horizontal red line indicates the median, and whiskers extend to 1.5 × IQR. No statistically significant differences were observed across sampling time points (*p* > 0.05).

Boxplots of urinary MA and PGA (Figure 2) showed fluctuations across the study period, but no significant differences were observed between baseline, day 6, and day 11 (*p* = 0.845 and *p* = 0.737, respectively). Detection rates for these metabolites remained stable, ranging from approximately 75% to 80% of samples. Salivary MDA also displayed a modest upward trend over time, though differences were not statistically significant (*p* = 0.595). The median and mean values followed a similar pattern, with slightly higher concentrations at days 6 and 11 compared with baseline. We also examined the combined sum Σ(MA + PGA), detailed in Table S1, for comparison with regulatory benchmarks.

### 3.4. Comet Assay

**Figure 3:**
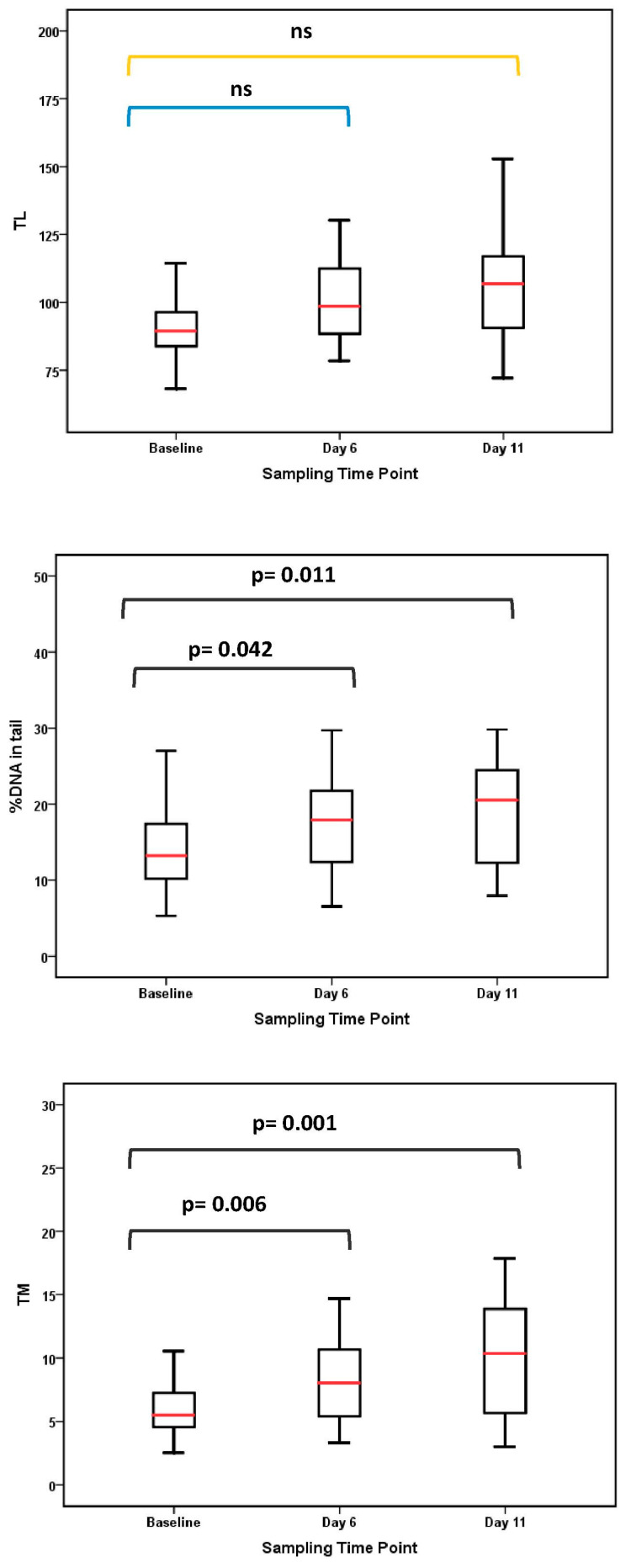

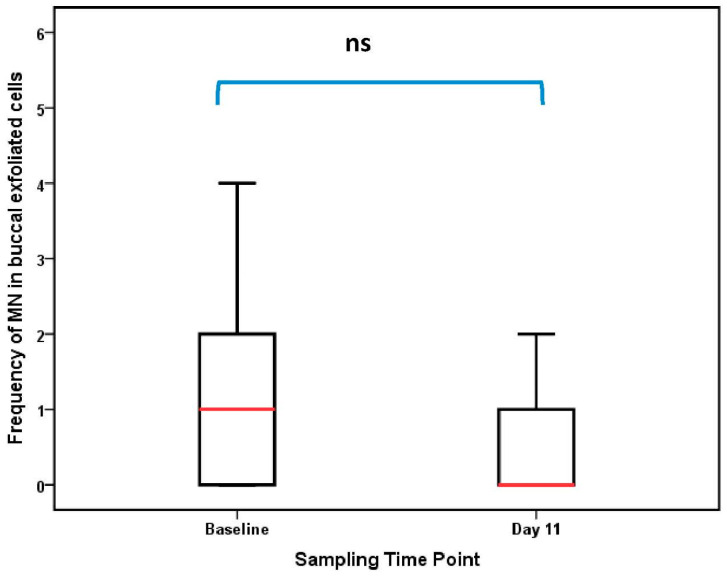
Boxplots showing comet assay indicators [tail length (TL), tail intensity (%DNA in tail), and tail moment (TM)] in peripheral blood lymphocytes and micronucleus (MN) frequency in exfoliated buccal cells at baseline, day 6, and day 11; MN frequency was assessed at baseline and day 11 only. Boxes represent the interquartile range (IQR), the horizontal red line within each box indicates the median, and whiskers extend to 1.5 × IQR. Statistical comparisons are shown where applicable; ns indicates *p* > 0.05. Blue and yellow “ns” labels indicate different pairwise comparisons and do not represent different levels of statistical significance.

**Figure 4:**
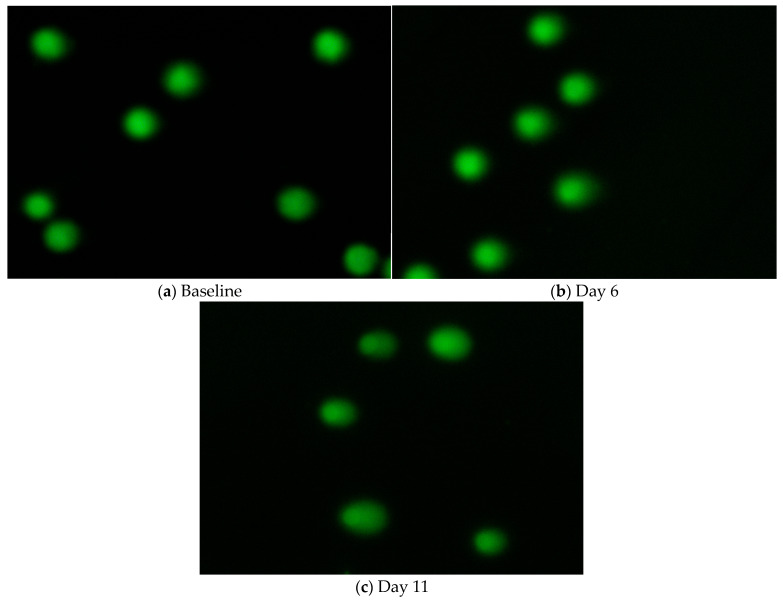
Representative comet images from Participant 24 at (**a**) baseline (minimal comet tailing; TM = 4.0, arbitrary units), (**b**) day 6 (moderate comet tailing; TM = 9.7, arbitrary units), and (**c**) day 11 (more pronounced comet tailing; TM = 15.5, arbitrary units).

Boxplots for comet assay parameters (Figure 3) showed increasing trends across the exposure period, particularly for %DNA in tail and TM. Descriptive percentage changes based on estimated marginal means are presented in Table 2. Representative comet images from a single donor (Figure 4) illustrate this trajectory: intact nuclei with minimal tailing at baseline, moderate DNA migration at day 6, and pronounced comet tails with extensive fragmentation by day 11. Descriptive statistics for comet assay parameters are presented in Supplementary Table S1.

### 3.5. Micronucleus Assay

The MN frequency in buccal cells remained low throughout the study and did not differ significantly between baseline and day 11 (*p* = 0.341). Boxplots (Figure 3) show median MN frequency of 1 per 1000 cells (range: 0–6) at baseline and 0.0 (range: 0–6) at day 11. MN frequencies > 1 per 1000 cells were observed in 11 participants (28.2%) at baseline and 8 participants (20.5%) at day 11. No consistent pattern was observed among participants with higher MN frequency (>3 per 1000 cells). Complete descriptive statistics for MN frequency are provided in Supplementary Table S1.

### 3.6. Selection of Covariates

Spearman’s rank correlation analysis was performed to assess associations between continuous participant characteristics and baseline biomarkers. Several anthropometric measures showed consistent negative relationships with comet assay indicators. Body weight was inversely correlated with %DNA in tail (rs = −0.448, *p* = 0.009) and TM (rs = −0.498, *p* = 0.003), while body height was inversely correlated with %DNA (rs = −0.418, *p* = 0.015) and TM (rs = −0.406, *p* = 0.019). Body mass index (BMI) was negatively associated with TL (rs = −0.344, *p* = 0.050) and TM (rs = −0.346, *p* = 0.048). These results suggest that body size parameters may influence DNA damage as detected by the comet assay.

The duration of residence in the same area was inversely correlated with urinary MA (r = −0.365, *p* = 0.024) but positively correlated with MN frequency (r = 0.373, *p* = 0.025), indicating possible cumulative environmental effects. Cotinine concentrations were not significantly associated with any of the biomarkers.

The Mann–Whitney U test was used to evaluate categorical predictors. Sex showed significant differences in %DNA (*p* = 0.014) and TM (*p* = 0.038), with males exhibiting higher DNA damage. Marital status was associated with MN frequency (*p* = 0.018), and education level was linked to TL (*p* = 0.037). Work status was significantly associated with salivary MDA (*p* = 0.027). Living in mixed residential areas was associated with higher salivary MDA (*p* = 0.009) and longer TL (*p* = 0.024). The use of Styrofoam cups was associated with greater TL (*p* = 0.016) and higher MN frequency (*p* = 0.014). Finally, the number of cups consumed per day was strongly associated with urinary MA (*p* = 0.005), salivary MDA (*p* < 0.001), and both TL and %DNA (*p* < 0.001).

Taken together, these findings identify anthropometric (weight, height, BMI), environmental (duration of residence, residential type, Styrofoam use), and demographic (sex, marital status, education, work status) variables as significant covariates for multivariable modeling of biomarker outcomes. Results are presented in Table S2.

### 3.7. Multivariable Analysis

Generalized estimating equation (GEE) models, adjusted for age, sex, body weight, and the number of tea or coffee cups consumed daily, showed higher comet assay values at follow-up compared with baseline, particularly for %DNA in the tail and TM (Table 2). TM, the prespecified primary comet assay endpoint, was significantly elevated at both follow-up time points compared with baseline (day 6: β = −0.438, 95% CI: −0.781 to −0.096, *p* = 0.006; day 11: β = −0.507, 95% CI: −0.846 to −0.168, *p* = 0.001; both Bonferroni-adjusted).

Secondary descriptive parameters showed similar trends, with significant increases in %DNA in tail at days 6 and 11 (day 6: β = −0.320, *p* = 0.042; day 11: β = −0.367, *p* = 0.011), whereas changes in TL were small and reached only borderline significance at day 11 (*p* = 0.091). Relative to baseline, descriptive percentage increases were 28.6% and 33.06% for TM, 13.39% and 15.37% for %DNA in tail, and 2.24% and 2.48% for TL at days 6 and 11, respectively. These percentages were calculated from estimated marginal means and are presented only to aid interpretation.

In addition to temporal effects, higher comet assay values were observed among participants consuming two cups per day compared with one cup per day. Participants who consumed two cups per day exhibited significantly higher TM levels than those who consumed one cup per day (β = 0.491, 95% CI: 0.219 to 0.764, *p* < 0.001). Similar exposure-related patterns were seen for the secondary indicators [%DNA in tail: β = 0.347, 95% CI: 0.095 to 0.600, *p* = 0.007; TL: β = 0.218, 95% CI: 0.127 to 0.309, *p* < 0.001]. Descriptively, the two-cup group showed 30.63% higher TM, 14.22% higher %DNA in tail, and 4.92% higher TL compared with the one-cup group. These findings suggest stronger comet assay responses among participants with higher daily cup consumption.

In addition to comet assay endpoints, secondary outcomes, including urinary styrene metabolites [MA, PGA, and Σ(MA + PGA)], salivary MDA, and MN frequency, were assessed using GEE models but did not show significant temporal changes. To enhance transparency, these non-significant temporal models are summarized in Supplementary Table S3. Percentage changes were not reported for MDA and MN because negative or very low estimated marginal means can produce unstable or potentially misleading relative values; therefore, these outcomes are presented using model-based estimates only. Although relative percentage changes were observed for some secondary outcomes, none of the temporal changes in urinary MA, PGA, Σ(MA + PGA), salivary MDA, or MN frequency reached statistical significance. Between-group comparisons showed higher MA and salivary MDA levels among participants consuming two cups per day compared with one cup per day; however, these findings should be interpreted cautiously because they were not accompanied by consistent temporal increases.

## 4. Discussion

This single-arm longitudinal human biomonitoring study suggests that short-term consumption of hot beverages in polystyrene cups may be associated with measurable styrene migration under consumer-use conditions and modest changes in selected early biomarkers of biological effect. Although styrene migration into beverages remained below the WHO guideline value, repeated exposure over the study period was associated with progressive increases in comet assay indicators, particularly TM and %DNA in tail. Based on measured migration levels and a standardized cup volume, estimated styrene intake ranged from 0.93 to 1.45 μg/day for one cup and 1.86–2.89 μg/day for two cups. These findings provide preliminary human data on repeated low-level exposure from food-contact materials and support further investigation of biologically relevant responses in real-world consumer-use scenarios.

The urinary styrene metabolites MA and PGA were detected consistently throughout the study period. However, they exhibited wide interindividual variability and no consistent time trend, reflecting their limited specificity and susceptibility to confounding by co-exposures such as other aromatic solvents, dietary constituents, and smoking-related pollutants [16,32,48]. Such variability is a well-recognized feature of population biomonitoring data derived from spot urine samples and may reflect differences in exposure timing, elimination kinetics, and metabolic half-life relative to sampling time rather than true differences in long-term exposure magnitude [49]. Importantly, the use of a within-subject repeated-measures design and GEE approach allowed us to account for correlated observations and focus on temporal changes within individuals, thereby reducing the influence of between-subject variability on the primary analyses [50].

Because occupational benchmarks such as the ACGIH Biological Exposure Index (BEI; 400 mg/g creatinine at the end of the shift) [51] and the German Biological Tolerance Value (400–600 mg/g creatinine) [15] are defined for the sum of MA and PGA Σ(MA + PGA), we evaluated both single and combined metabolite concentrations, expressed as raw values and creatinine-adjusted values (Table S1). On average, urinary metabolite levels remained well below the BEI; however, a subset of participants exceeded this threshold—6 at baseline, 3 at day 6, and 8 at day 11. The fact that exceedances occurred even in a short-term, consumer-level exposure setting highlights interindividual variability in internal dose under consumer-use conditions and may reflect differences in susceptibility within the general population (Table S4). This variability is consistent with occupational biomonitoring studies [48]. For example, workers in the fiberglass-reinforced plastic and cured-in-place pipe lining sectors have shown Σ(MA + PGA) levels > 1000 mg/g creatinine despite lower group averages [12]. Similarly, Italian FRP workers exhibited medians ranging from 7.3 to 331.1 mg/g creatinine, influenced by process type, co-exposure to acetone, and the use of respiratory protection [32]. In contrast, US population-based biomonitoring (NHANES 2005–2006 and 2011–2012) reported median creatinine-adjusted concentrations of ~121–124 μg/g for MA and 164–173 μg/g for PGA among non-users, with about twofold higher levels in smokers [52]. These general population levels are well below occupational BEIs but overlap with the lower end of occupational exposure levels, highlighting the importance of accounting for variability in susceptibility.

Secondary outcomes (urinary metabolites, MDA, and MN frequency) did not change significantly during the intervention, consistent with prior reports that these markers are less sensitive to short-term, low-level styrene exposure than comet assay endpoints. Similarly, salivary MDA showed only modest, non-significant increases, consistent with evidence that lipid peroxidation markers are not highly specific, as they may also be elevated by unrelated environmental exposures, diet, or passive smoking [36].

By contrast, comet assay indicators showed consistent upward changes over time, with stronger responses among participants consuming two cups daily. In this context, the comet assay may have been more sensitive than the MN assay for capturing short-term changes under the present exposure scenario. Tail length, %DNA in tail, and particularly TM all increased significantly with repeated exposure, with stronger effects among participants consuming two cups daily. This pattern is biologically plausible, given that styrene is metabolized to styrene-7,8-oxide, a DNA-reactive epoxide that has been linked to strand breaks and adduct formation [18]. The MN assay, however, did not show significant changes, which may reflect the limited sensitivity of buccal MN frequency for short-term, low-level exposures and the longer turnover time of buccal epithelial cells [53]. Although the absolute effect sizes were modest, expressing changes relative to baseline highlighted a consistent temporal pattern. TM increased descriptively by 28.6% at day 6 and 33.06% at day 11, while %DNA in tail increased by 13.39% and 15.37%, respectively. TL showed smaller descriptive increases of 2.24% at day 6 and 2.48% at day 11, consistent with its lower sensitivity as an endpoint compared with TM and %DNA in the tail. Accordingly, the observed comet assay changes should be interpreted as non-specific indicators of early biological effect rather than as evidence of clinically meaningful or styrene-specific genotoxic risk.

Importantly, the lack of concordance across biomarkers warrants careful interpretation. Although comet assay parameters showed consistent increases over time, urinary MA and PGA, salivary MDA, and micronucleus frequency did not demonstrate significant temporal changes. This apparent inconsistency may reflect differences in the sensitivity, specificity, kinetics, and biological relevance of these biomarkers. The comet assay is a sensitive indicator of DNA strand breaks but is not specific to styrene exposure and may respond to a range of endogenous and exogenous stressors. In contrast, urinary MA and PGA may be influenced by exposure timing, metabolism, spot-urine variability, and background environmental sources. Similarly, MDA and buccal MN frequency may be less responsive to short-term, low-level exposure over the present follow-up period. Therefore, the observed comet assay findings should be interpreted as preliminary, non-specific early biomarkers of biological effect, and not as definitive evidence of styrene-specific genotoxicity.

Comparable findings have been reported in occupational cohorts, where styrene-exposed workers demonstrated significantly elevated single- and double-strand DNA breaks, along with reduced DNA repair activity, in comet assays; notably, workplace styrene concentrations correlated with comet parameters, while urinary Σ(MA + PGA) showed inverse associations with oxidative DNA damage [54]. In line with this, Laffon et al. [55] observed that styrene-exposed fiberglass workers had significantly higher SCE and MN frequencies, as well as increased comet TL compared with controls, reinforcing the utility of these biomarkers in assessing genotoxicity. Inter-individual variability in genotoxic outcomes may also reflect differences in DNA repair capacity. In this regard, Slyskova et al. [56] reported that styrene-exposed lamination workers showed moderate but variable repair of oxidative DNA damage, which was influenced by smoking status, sex, and genetic polymorphisms in DNA repair genes (GSTM1, XRCC1, XPC). These findings underscore that individual susceptibility, in addition to exposure intensity, may shape genotoxic responses to styrene. In addition, styrene-related oxidative and carbonyl stress pathways, including the formation of advanced glycation end products (AGEs), may contribute to DNA damage responses; however, AGE formation and DNA glycation were not directly measured in the present study and should therefore be considered only as plausible mechanistic pathways requiring further investigation [57].

Notably, we did not find consistent associations between urinary styrene metabolites [MA, PGA, or Σ(MA + PGA)] and comet parameters. Yet, progressive changes in comet assay indicators were evident over time and were accentuated in the two-cup group. This discrepancy suggests that additional compounds migrating from Styrofoam cups, beyond styrene itself, may have contributed to the observed biological responses. Polystyrene food-contact materials may contain or release not only styrene monomer but also low-molecular-weight oligomers, additives, and other co-leached substances, which may contribute to biological responses either independently or synergistically and warrant further investigation in future studies [58]. In addition, co-monomers and packaging-related additives, including compounds such as bisphenols, may further contribute to oxidative stress and genotoxic responses through overlapping or interacting mechanisms [59]. Co-exposures from plastic-derived contaminants, additives, combustion-related pollutants, and other environmental chemicals may therefore modify or amplify these responses, limiting the specificity of the observed biomarker changes to styrene alone. It is also essential to consider that consumer exposures rarely involve styrene in isolation. Experimental work on styrene–butadiene mixtures has revealed distinct genotoxic patterns compared to single compounds, with cross-linking and oxidative mechanisms driven, in part, by butadiene diepoxide [60]. Such findings underscore the complexity of co-exposures and suggest that the DNA damage observed in our study may similarly reflect the contribution of other leachable chemicals from Styrofoam cups rather than styrene alone. In this context, recent occupational and experimental evidence indicates that styrene may not act alone. A study of workers exposed to a styrene–xylene mixture found higher frequencies of nuclear buds and oxidative DNA damage, even though air levels were below occupational limits. In vitro experiments with human blood cells confirmed that the mixture induced micronuclei and nuclear buds more strongly than either compound alone, suggesting additive or synergistic effects [61]. These findings underscore that regulatory thresholds based on single compounds may underestimate risks in real-world co-exposure scenarios.

Our findings align with occupational studies, which show complex biomarker responses to styrene exposure. For example, a biomonitoring study of fiberglass-reinforced plastic workers reported higher urinary MA + PGA levels and increased MN frequency in open-molding processes, but found oxidative rather than direct DNA damage, as indicated by the Fpg-comet assay and urinary oxidized guanine, which emerged as sensitive biomarkers of oxidative stress [62]. Similarly, the EFSA Panel on Food Contact Materials recently concluded that oral exposure to styrene did not induce genotoxic effects in reliable in vivo studies, and that migration of styrene at or below the Specific Migration Limit of 40 μg/kg food does not raise safety concerns [63]. However, differences in study design, exposure route, duration, and evaluated endpoints may partly explain discrepancies between controlled animal studies, regulatory assessments, and short-term human biomonitoring findings. A 28-day oral gavage study in Fischer 344 rats using Organization for Economic Co-operation and Development -compliant assays (comet, micronucleus, Pig-a) likewise reported no styrene-induced genotoxicity at doses up to 500 mg/kg/day [64].

Strengths of this study include its prospective longitudinal design; the simultaneous assessment of multiple biomarker endpoints; and the application of generalized estimating equations with a Bonferroni correction to appropriately account for repeated measures and multiple testing. Another strength is that the participants were hospital employees, a relatively homogenous population with good compliance and low attrition, which reduced variability unrelated to exposure and enhanced the internal validity of the findings.

Nevertheless, several limitations must be acknowledged. The relatively small sample size limited statistical power, particularly for secondary outcomes (urinary metabolites, salivary MDA, and MN frequency), and constrained the ability to adjust analyses for specific confounders [65]. In addition, 1000 differentiated buccal epithelial cells per participant were scored for the micronucleus assay, whereas the HUMNxL protocol recommends scoring up to 2000 cells to maximize sensitivity [42]. Although 1000 cells have been widely applied in human biomonitoring studies, the use of a higher cell count may increase the ability to detect subtle changes in MN frequency. Moreover, the intervention period was relatively short, making it challenging to capture longer-term biological responses. For the buccal MN assay, sampling at day 11 may have been too early to fully capture exposure-related MN formation, because buccal epithelial cell turnover can extend up to 21–28 days, and recent recommendations suggest that later sampling, particularly ≥21 days after exposure, may be more informative [46]. Therefore, the day 11 MN result may partly reflect exposures occurring before or during the washout period rather than the full intervention effect, and the null MN finding should be interpreted cautiously. Several methodological limitations should also be noted. Adherence to the washout and intervention procedures was assessed based on participant instructions and self-report, rather than on direct observation or daily exposure diaries. No a priori power calculation was performed; therefore, the findings should be interpreted as exploratory and hypothesis-generating rather than confirmatory. The comet assay was based on 50 cells per participant per time point; although %DNA in tail was reported as a key comet assay descriptor, this cell count is lower than the ≥100 cells recommended by some recent protocols and may have reduced assay sensitivity and reproducibility. In addition, styrene migration was assessed using a standardized 60 min contact time, which represents a conservative exposure scenario and may overestimate typical consumer exposure, as actual drinking duration, individual contact time, and precise beverage temperature were not quantified. In addition, the analytical approach focused on targeted quantification of styrene and selected biomarkers using validated gas chromatography–mass spectrometry (GC–MS) and high-performance liquid chromatography (HPLC)-based methods; complementary spectroscopic techniques such as attenuated total reflectance–Fourier transform infrared spectroscopy (ATR-FTIR) or near-infrared spectroscopy (NIR) were not included and could be considered in future studies to better characterize additional monomers, additives, or polymer-derived components. Although assessment of styrene-7,8-oxide formation and styrene-derived DNA adducts was originally planned, these analyses could not be completed due to instrumental failure of the GC–MS system required for their measurement and the unavailability of alternative facilities within the study timeframe. Consequently, DNA adducts—which would have provided the most specific evidence of styrene–DNA interactions—could not be obtained. While DNA adduct analysis would have provided more direct mechanistic evidence of styrene–DNA interaction, such data were not available in the present study. Therefore, the observed longitudinal changes in comet assay indicators should be interpreted as preliminary evidence of early biological response rather than definitive proof of styrene-specific genotoxicity. In addition, the observed changes cannot be attributed exclusively to styrene monomer. Previous studies have demonstrated that polystyrene food-contact materials may release not only styrene but also low-molecular-weight oligomers, polymer fragments, and microplastic particles, particularly under elevated temperature and prolonged contact conditions. These co-leached constituents may contribute to oxidative stress and DNA damage either independently or synergistically with styrene [66].

Background exposures, such as secondhand smoke (as indicated by detectable urinary cotinine in a subset of participants), urban air pollution, and other sources of styrene (e.g., contact with polystyrene food packaging), may have influenced urinary metabolites or oxidative stress markers, thereby reducing the specificity of these markers for styrene alone. A further limitation is the absence of a parallel comparator group using inert materials such as glass, ceramic, or stainless steel. Because both tea and coffee were included to reflect real-world hot beverage consumption, differences in the beverage matrix may have influenced styrene migration and should be controlled more strictly in future studies. Although the within-subject baseline-to-follow-up design reduced between-person variability, it could not eliminate residual environmental exposures or fully distinguish effects related to polystyrene cup use from temporal or background exposure patterns. In addition, individual exposure was not directly quantified. Styrene migration testing was performed using the same cup type used during the intervention and provided an estimate of potential exposure under standardized conditions; however, it did not capture individual-level variation in beverage temperature, actual contact duration, drinking behavior, inhalation exposure, diet, ambient air, or other background sources. These limitations constrain mechanistic interpretation and should be addressed in future studies using parallel inert-cup control groups and direct individual exposure assessment. Residual variability may also reflect unmeasured factors such as genetic differences in styrene metabolism and dietary sources, which may have contributed to non-specific biomarker responses in this real-world consumer exposure setting [49]. Participants with detectable urinary cotinine were not excluded from the main analysis, as this likely reflected possible secondhand smoke exposure rather than confirmed active smoking; however, this remains a potential source of residual confounding. While it was not feasible to fully control all dietary or environmental exposures during the intervention period in a real-world human study, the within-subject repeated-measures design allowed each participant to serve as their own control, thereby minimizing the influence of stable individual behaviors and background exposures over the 14-day period [50,67]. Participants were not restricted from consuming other foods or beverages packaged in plastic materials during the intervention period; therefore, background dietary exposure to styrene from non-study sources cannot be excluded and may have contributed to biomarker variability. Occasional or rare use of Styrofoam for food storage reported by a small number of participants was not modeled as a primary exposure variable due to its infrequent nature but is acknowledged as a potential source of residual styrene exposure. Styrene migration was assessed using a conservative 60 min hot beverage contact time and a single Styrofoam cup brand, which may overestimate real-world consumer exposure and limit the generalizability of the migration findings to other polystyrene products. Migration studies have shown that prolonged contact times and standardized testing conditions can exaggerate apparent migration relative to typical food-use scenarios due to polymer–simulant interactions [68]. In addition, beverage temperature was not instrumentally controlled, and differences in beverage composition and cooling profiles between tea and coffee may have influenced styrene migration despite standardized handling conditions. Finally, because participants were recruited from a workplace setting and consisted primarily of hospital employees, the findings may not be fully generalizable to the broader population. In addition, the lack of concurrent environmental exposure measurements limits the ability to distinguish the contribution of co-exposures known to influence oxidative stress and comet assay–related biomarker responses [69]. This may also include emerging plastic-derived contaminants, such as micro- and nanoplastics, which may enter the human body through ingestion and have been linked to oxidative stress and inflammatory responses [70].

Despite these limitations, our findings suggest that everyday consumer exposure to styrene-containing food-contact materials may be associated with measurable changes in selected early biomarkers of biological effect. While current regulatory limits are based mainly on migration levels, our results indicate that low-level, repeated exposures were associated with measurable changes in selected response markers under the conditions of this study. Future research should include larger populations, longer follow-up durations, and the integration of more specific endpoints—such as DNA adducts, styrene-7,8-oxide quantification, and omics-based biomarkers—to better disentangle the contribution of styrene from other pollutants and enhance mechanistic understanding and causal interpretation.

## 5. Conclusions

This single-arm longitudinal human biomonitoring study provides preliminary evidence that repeated short-term consumption of hot beverages in polystyrene cups may be associated with modest changes in selected early biomarkers of biological effect under consumer-use conditions. While styrene migration remained below the WHO guideline value, comet assay indicators increased over the exposure period, particularly among participants with higher cup consumption. These findings should be interpreted cautiously due to the modest sample size, short duration, and the lack of more specific mechanistic endpoints. Nevertheless, the study highlights the relevance of evaluating repeated low-level exposure to food-contact materials and supports larger studies with longer follow-up and more specific exposure and response biomarkers.

## Figures and Tables

**Table 1:** Baseline characteristics of 41 participants.

	Mean	SD	Median	Minimum	Maximum
Age	31.2	5.7	30.8	21.9	41.8
Weight (kg)	65.5	16.2	63.0	31.2	122.0
Height (m)	1.61	0.09	1.59	1.49	1.80
BMI (kg/m^2^)	25.1	4.7	25.3	13.7	37.7
Duration of living in the residence area (years)	7.6	7.8	6.0	0.3	30.0
Cotinine in urine (μg/L)	17.2	48.8	0.8	0.0	215.2
**Categorical variables**				**Count/(percentage %)**	
Sex	Male/Female			12 (29.3)/29 (70.7)	
Marital status	Married/Single			17 (41.5)/24 (58.5)	
Educational level	≤High school/>High school			14 (34.1)/27 (65.9)	
Work status	Yes/No			32 (78)/9 (22)	
Income (Saudi Riyals)	<5000/5000–10,000/>10,000/Refused/No income			15 (36.6)/4 (9.8)/6 (14.6)/6 (14.6)/8 (19.5)	
Conditions of the residential area	Clean/Mixed (polluted and clean)			28 (68.3)/13 (31.7)	
Use of Styrofoam cups for drinking	Yes/No			29 (70.7)/12 (29.3)	
Frequency of Styrofoam cups usage	Daily/Weekly/Monthly (seldom)			13 (31.7)/7 (17.1)/9 (22)	
Type of drinking	Hot beverages/Cold beverages/Both			22 (53.7)/3 (7.3)/4 (9.8)	
Use of Styrofoam cups for storing food	Yes/No			6 (14.6)/35 (85.4)	
Living with smokers	Yes/No			10 (24.4)/31 (75.6)	
Socialize with smokers	Yes/No			23 (56.1)/18 (43.9)	
Frequency of coffee/tea consumption during the study	Once/Twice			19 (46.3)/22 (53.7)	
Type of hot beverage consumed during the study	Tea/Coffee/Both			10 (24.4)/24 (58.5)/7 (17.1)	

Footnote: Sample size varies by variable due to missing data. Baseline demographic characteristics were available for up to 41 participants; anthropometric measures (weight, height, BMI, urinary cotinine) were available for 40 participants, and duration of residence data were available for 38 participants.

**Table 2:** Pairwise comparisons of estimated marginal means for comet assay parameters. Values represent β coefficients (mean differences) with standard errors, 95% confidence intervals, Bonferroni-adjusted *p*-values, and descriptive percentage changes/differences calculated from estimated marginal means.

Outcome	Comparison	β (Mean Difference)	SE	95% CI	% Change/Difference	*p*-Value
TL	Baseline vs. Day 6	−0.100	0.050	−0.221, 0.021	+2.24%	0.142
	Baseline vs. Day 11	−0.111	0.051	−0.233, 0.012	+2.48%	0.091
	2 cups vs. 1 cup	0.218	0.046	0.127, 0.309	+4.92%	<0.001
Tail Intensity (% DNA in tail)	Baseline vs. Day 6	−0.320	0.130	−0.630, −0.009	+13.39%	0.042
	Baseline vs. Day 11	−0.367	0.127	−0.670, −0.064	+15.37%	0.011
	2 cups vs. 1 cup	0.347	0.129	0.095, 0.600	+14.22%	0.007
TM	Baseline vs. Day 6	−0.438	0.143	−0.781, −0.096	+28.6%	0.006
	Baseline vs. Day 11	−0.507	0.142	−0.846, −0.168	+33.06%	0.001
	2 cups vs. 1 cup	0.491	0.139	0.219, 0.764	+30.63%	<0.001

Footnote: Pairwise comparisons were performed using generalized estimating equations (GEE) with Bonferroni correction. Percentage changes/differences were calculated descriptively from estimated marginal means as [(follow-up or two-cup value − reference value)/reference value] × 100 and are provided to aid interpretation. Negative β coefficients in baseline comparisons indicate higher values at follow-up compared with baseline due to model coding. Positive β coefficients for the two-cup comparison indicate higher values among participants consuming two cups per day compared with one cup per day. TL, tail length reported in μm; Tail intensity (%DNA in tail) is reported as percentage DNA in tail; TM, tail moment reported as arbitrary units.

## Data Availability

The data presented in this study are available on request from the corresponding author due to privacy and ethical restrictions related to human participant data.

## Supplementary Materials

The following supporting information can be downloaded at: https://www.mdpi.com/article/10.3390/jox16030084/s1. Table S1: Urinary styrene metabolites mandelic acid (MA) and phenylglyoxylic acid (PGA), salivary malondialdehyde (MDA), comet assay parameters, and micronucleus frequency at baseline, day 6, and day 11 in 41 volunteers; Table S2: Bivariate analyses of baseline variables with urinary styrene metabolites (MA and PGA), salivary MDA, comet assay parameters, and MN frequency. Spearman’s rank correlation was used for continuous variables (rs and p shown), and the Mann–Whitney U test for categorical variables (p shown); Table S3: Generalized estimating equation (GEE) models for secondary outcomes, including urinary styrene metabolites, salivary MDA, and micronucleus frequency; Table S4: Comparison of urinary styrene metabolite (MA and PGA) levels with regulatory benchmarks and general population data. References [15,16,32,51,52] are cited in the Supplementary Materials.
