# Supplementary material for: Short-Term Consumption of Hot Beverages in Polystyrene Cups and Early Biomarkers of Biological Effect: A Single-Arm Longitudinal Human Biomonitoring Pilot Study
Source: J Xenobiot. 2026 May 15;16(3):84. doi: 10.3390/jox16030084 (PMC13214965; doi:10.3390/jox16030084)
Supplement: Supplementary file 1 [file jox-16-00084-s001.zip › jox-4298413-supplementary.pdf]

Journal of Xenobiotics

Supplementary Materials: Short-Term Consumption of Hot Beverages in Polystyrene Cups and Early Biomarkers of Biological Effect: A Single-Arm Longitudinal Human Biomonitoring Pilot Study

Iman Al-Saleh, Ghofran Al-Qudaihi, Yara Aljerayed, Kafa Abuhdeeb, Rola Elkhatib, Hissah Alnuwaysir, Mashael Alsubaie and Norah Alotaibi

Table S1. Urinary styrene metabolites mandelic acid (MA) and phenylglyoxylic acid (PGA), salivary malondialdehyde (MDA), comet assay parameters, and micronucleus frequency at baseline, day 6, and day 11 in 41 volunteers

Biomarker	Baseline			Day 6			Day 11			p-value
	n	Mean	Median, Range	n	Mean	Median, Range	n	Mean	Median, Range	
MA (mg/L)	41	78.7	33.8 (0–933.1)	41	81.7	34.3 (0–640.4)	41	81.0	41.3 (0–580.8)	0.845
MA (mg/g creatinine)	41	167.9	29.2 (0–2266)	41	163.9	37.0 (0–3887.6)	41	170.3	64.2 (0–3048.4)	0.365
PGA (mg/L)	41	121.0	60.6 (0–885.3)	41	77.2	44.7 (0–389.0)	41	104.4	41.6 (0–831.0)	0.737
PGA (mg/g creatinine)	41	221.1	56.6 (0–4531.8)	41	113.4	56.2 (0–908.3)	41	156.8	55.8 (0–1807.2)	0.983
Σ(MA + PGA), (mg/L)	41	199.7	119.0 (0–933.1)	41	158.9	133.2 (0–790.0)	41	185.4	110.8 (0–946.0)	0.809
Σ(MA + PGA), (mg/g creatinine)	41	389.1	145.1 (0–4531.8)	41	277.3	165.1 (0–4795.9)	41	327.0	139.3 (0–3048.4)	0.911
Salivary MDA (nmol/mL)	35	0.59	0.51 (0.25–1.85)	35	0.65	0.54 (0.02–1.89)	35	0.64	0.55 (0.34–2.6)	0.595
TL (μm)	34	94.5	89.4 (68.1–140.7)	34	101.5	98.5 (78.5–163.2)	34	105.5	106.8 (72.1–152.8)	0.042
Tail intensity (% DNA in tail)	34	14.1	13.2 (5.3–31.0)	34	17.9	17.9 (6.6–38.4)	34	19.3	20.6 (8.0–29.8)	0.004
TM	34	6.6	5.6 (2.5–16.4)	34	8.8	8.0 (3.3–26.7)	34	10.0	10.4 (3.0–17.8)	0.002
MN frequency (per 1,000 cells)	39	1.2	1.0 (0–6)		-	Not assessed	39	0.9	0.0 (0–6)	0.341

Footnote: Sample sizes vary across biomarkers due to missing data or insufficient biological material. All available data were included for each outcome. P-values were derived from generalized estimating equation (GEE) models for repeated measures; MN analysis compares baseline vs. day 11 only.

https://doi.org/10.3390/jox16030084

Table S2. Bivariate analyses of baseline variables with urinary styrene metabolites (MA and PGA), salivary MDA, comet assay parameters, and MN frequency. Spearman's rank correlation was used for continuous variables (rs and p shown), and the Mann–Whitney U test for categorical variables (p shown).

Continuous variables		MA	PGA	MDA	TL	%DNA in tail	TM	MN frequency
Age	rs	0.031	-0.181	0.305	0.310	0.015	0.097	-0.209
	p	0.847	0.259	0.075	0.074	0.934	0.585	0.201
	n	41	41	35	34	34	34	39
Weight (kg)	rs	0.103	-0.100	-0.194	-0.290	**-0.448****	**-0.498****	-0.075
	p	0.527	0.540	0.271	0.102	**0.009**	**0.003**	0.653
	n	40	40	34	33	**33**	**33**	38
Height (meters)	rs	0.199	0.151	-0.294	-0.133	**-0.418***	**-0.406***	-0.084
	p	0.219	0.353	0.092	0.461	**0.015**	**0.019**	0.615
	n	40	40	34	33	**33**	**33**	38
BMI (kg/m^2)	rs	-0.092	-0.146	-0.182	-0.344	-0.274	**-0.346***	0.021
	p	0.574	0.368	0.302	0.050	0.123	**0.048**	0.900
	n	40	40	34	33	33	**33**	38
Duration of living in the area in years	rs	**-0.365***	0.021	-0.083	0.055	0.120	0.086	**0.373***
	p	**0.024**	0.899	0.651	0.770	0.519	0.647	**0.025**
	n	**38**	38	32	31	31	31	**36**
Cotinine (µg/L)	rs	-0.010	-0.047	0.104	0.148	-0.059	0.001	0.167
	p	0.951	0.774	0.558	0.404	0.740	0.996	0.318
	n	40	40	34	34	34	34	38
**Categorical variables**		**MA**	**PGA**	**MDA**	**TL**	**%DNA in tail**	**TM**	**MN frequency**
Sex (Females/Males)	p	0.535	0.326	0.477	0.850	**0.014**	**0.038**	0.283
Marital status (Single/Married)	p	0.915	0.679	0.549	0.567	0.059	0.074	**0.018**
Educational level (<= 12 years/ >12 years)	p	0.967	0.293	0.154	**0.037**	0.387	0.564	0.525
Work status (Yes/No)	p	0.865	0.816	**0.027**	0.071	1.000	0.925	0.857
Living in residential (Clean/ Mixed)	p	0.446	0.214	**0.009**	**0.024**	0.986	0.790	0.404
Living with smokers (Yes/No)	p	0.951	0.264	0.875	0.850	0.427	0.427	0.324
Socialize with smokers (Yes/No)	p	0.420	0.383	0.301	0.736	0.632	0.929	0.976
Use of Styrofoam cups (Yes/No)	p	0.162	0.965	0.970	**0.016**	0.151	0.089	**0.014**
Storing food in Styrofoam containers (Yes/No)	p	0.376	0.957	0.949	0.775	0.878	0.947	0.951
Number of cups taken per study (one cup/two cups)	p	**0.005**	0.519	**0.000**	**0.000**	0.801	0.665	0.491

Footnote: Sample sizes (n) vary across biomarkers due to missing data or insufficient biological material. *$p < 0.05$; **$p < 0.01$.

Table S3. Generalized estimating equation (GEE) models for secondary outcomes, including urinary styrene metabolites, salivary MDA, and micronucleus frequency.

Outcome	Comparison	β (Mean Difference)	SE	95% CI	% Change/Difference	p-value
MA	Baseline vs Day 6	0.357	0.401	−0.602, 1.317	−8.16%	1.0
	Baseline vs Day 11	0.154	0.346	−0.675, 0.983	−3.52%	1.0
	2 cups vs 1 cup	0.918	0.365	0.204, 1.633	+24.52%	0.012
PGA	Baseline vs Day 6	−0.085	0.428	−1.111, 0.940	+2.13%	1.0
	Baseline vs Day 11	0.050	0.410	−0.931, 1.031	−1.25%	1.0
	2 cups vs 1 cup	0.089	0.429	−0.753, 0.930	+2.23%	0.836
Σ(MA + PGA)	Baseline vs Day 6	0.074	0.375	−0.824, 0.973	−1.56%	1.0
	Baseline vs Day 11	0.386	0.367	−0.494, 1.266	−8.09%	0.881
	2 cups vs 1 cup	0.593	0.385	−0.162, 1.349	+13.73%	0.124
MDA	Baseline vs Day 6	0.161	0.166	−0.235, 0.557		0.991
	Baseline vs Day 11	0.043	0.163	−0.348, 0.434		1.0
	2 cups vs 1 cup	0.433	0.188	0.064, 0.801		0.021
MN	Baseline vs Day 11	−0.217	0.504	−1.205, 0.771		0.666
	2 cups vs 1 cup	0.115	0.508	−0.881, 1.111		0.821

Footnote: Percentage changes/differences were calculated descriptively from estimated marginal means as [(follow-up or two-cup value − reference value) / reference value] × 100 and are provided only to aid interpretation. Statistical inference was based on GEE β coefficients, 95% CIs, and adjusted p-values. Percentage changes were not calculated for MDA because negative estimated marginal means limited meaningful interpretation of relative change, or for MN because very low baseline values produced unstable and potentially misleading relative percentages. MA, mandelic acid; PGA, phenylglyoxylic acid; MDA, malondialdehyde; MN, micronucleus frequency.

https://doi.org/10.3390/jox16030084

Table S4. Comparison of urinary styrene metabolite (MA and PGA) levels with regulatory benchmarks, occupational biomonitoring data, and general population data.

Source / Population	Biomarker(s)	Reported Level	Reference
Current study (Saudi Arabia, healthy volunteers, n=41; baseline median)	Σ(MA + PGA) (urine)	Median: 119 mg/L (145 mg/g creatinine)	This study
ACGIH BEI (occupational, end of shift)	Σ(MA + PGA) (urine, creatinine-adjusted)	400 mg/g creatinine	ACGIH (2015)
German BAT value (DFG)	Σ(MA + PGA) (urine)	400–600 mg/g creatinine	DFG (2021)
Italian FRP workers	Σ(MA + PGA), creatinine-adjusted urine	Median site values: 7.3–331.1 mg/g creatinine	Bonanni et al. (2015)
Volunteer inhalation study	Σ(MA + PGA) (urine)	Urinary levels comparable to 20–100 mg/L	Prieto et al. (2002)
U.S. general population (NHANES)	Σ(MA + PGA) (urine)	Low detectable levels <0.3 mg/g creatinine	Capella et al. (2019)

Footnote: MA = mandelic acid; PGA = phenylglyoxylic acid; BEI = Biological Exposure Index; BAT = Biologischer Arbeitsstoff-Toleranzwert (Biological Tolerance Value). Regulatory values apply to occupational exposure settings, whereas biomonitoring values reflect background exposures in the general population and are provided for contextual comparison only and are not intended as risk benchmarks for consumer exposure.

https://doi.org/10.3390/jox16030084

References:

1. ACGIH, 2015. Styrene: BEI® (Biological Exposure Indices®), American Conference of Governmental Industrial Hygienists Cincinnati, OH: ACGIH; 2015.
2. Bonanni, R.C., Gatto, M.P., Paci, E., Gordiani, A., Gherardi, M., Tranfo, G., 2015. Biomonitoring for exposure assessment to styrene in the fibreglass reinforced plastic industry: Determinants and interferents. Ann. Occup. Hyg. 59, 1000-1011.
3. Capella, K.M., Roland, K., Geldner, N., Rey deCastro, B., De Jesús, V.R., van Bemmel, D., Blount, B.C., 2019. Ethylbenzene and styrene exposure in the United States based on urinary mandelic acid and phenylglyoxylic acid: NHANES 2005-2006 and 2011-2012. Environ. Res. 171, 101-110.
4. DFG, 2021. Styrene: BAT Value Documentation, 2011. In: List of MAK and BAT Values 2021: Permanent Senate Commission for the Investigation of Health Hazards of Chemical Compounds in the Work Area. , in: Deutsche Forschungsgemeinschaft, G.R.F. (Ed.). Wiley-VCH, Weinheim, Germany.
5. Prieto, M.J., Marhuenda, D., Cardona, A., 2002. Analysis of styrene and its metabolites in blood and urine of workers exposed to both styrene and acetone. J. Anal. Toxicol. 26, 23-28.

https://doi.org/10.3390/jox16030084
